# Comparison of two techniques used in routine care for the treatment of inflammatory macular oedema, subconjunctival triamcinolone injection and intravitreal dexamethasone implant: medical and economic importance of this randomized controlled trial

**DOI:** 10.1186/s13063-020-4066-0

**Published:** 2020-02-10

**Authors:** Chloé Couret, Alexandra Poinas, Christelle Volteau, Valery-Pierre Riche, Marie-Laure Le Lez, Marie-Hélène Errera, Catherine Creuzot-Garcher, Stéphanie Baillif, Laurent Kodjikian, Catherine Ivan, Laurence Mathilde Le Jumeau de Kergaradec, Anne Chiffoleau, Alexandra Jobert, Julie Jaulin, Laetitia Biron, Elisabeth Hervouet, Michel Weber

**Affiliations:** 1 Department of Ophthalmology, CHU Nantes, Nantes, France; 2 Clinical Investigation Centre CIC1413, INSERM and CHU Nantes, Nantes, France; 3 Sponsor Department, Methodology and Biostatistics Platform, CHU Nantes, Nantes, France; 4 Research Department, Innovation Unit, CHU Nantes, Nantes, France; 5 Department of Ophthalmology, CHU Tours, Tours, France; 6 Quinze-Vingts National Ophthalmology Hospital, DHU Sight Restore, CIC 1423, Sorbonne-Universités, UPMC Université, Paris, France; 7 Department of Ophthalmology, CHU Dijon, Dijon, France; 8 Department of Ophthalmology, CHU Nice, Nice, France; 9 Department of Ophthalmology, Hospices Civils de Lyon, Lyon, France; 10 Sponsor Department, CHU Nantes, Nantes, France

**Keywords:** Macular oedema, Corticoids, Periocular injection, Intraocular injection, Medical cost–benefit analyses

## Abstract

**Background:**

Whether they are injected peri- or intraocularly, corticosteroids are still essential tools in the therapeutic arsenal for treating inflammatory macular oedema. A few years ago, however, only triamcinolone acetonide was available to ophthalmologists. While this compound was initially developed for rheumatological or dermatological use, it has been increasingly deployed in ophthalmology, despite still being off-label. In 2011, the system for delivery of dexamethasone from a biodegradable, injectable implant into the vitreous cavity obtained approval for use in inflammatory macular oedema. While the efficacy and safety of triamcinolone in macular oedema, including inflammatory oedema, have already been studied, there are currently no publications on subconjunctival triamcinolone injections, which are simple, effective and well tolerated. To date, the dexamethasone 700 μg implant has been authorized for the treatment of noninfectious intermediate and posterior uveitis, but there have been no studies to evaluate the efficacy and safety of the different peri- and intraocular strategies, including the treatment of inflammatory macular oedema.

**Methods:**

This protocol is therefore designed to compare the efficacy and safety of peri- and intraocular corticosteroid injections in the treatment of inflammatory macular oedema. In this ongoing study, 142 patients will be included, and the oedematous eye will be randomised to treatment with either subconjunctival triamcinolone injection or an intravitreal implant containing 700 μg dexamethasone. Follow-up is planned for 6 months with monthly visits. Each visit will include visual acuity measurement, a slit lamp examination, fundoscopy, intraocular pressure measurement, laser flare measurement (if available) and spectral domain optical coherence tomography.

**Discussion:**

The results of this trial will have a real impact on public health if it is shown that a Kenacort retard® (i.e. triamcinolone) injection costing just €2.84 and performed in the physician’s office (with no additional overhead costs) is at least as effective as the dexamethasone 700 μg implant (Ozurdex®; costing approximately €960 with the injection performed in a dedicated room), with no increased side effects.

**Trial registration:**

ClinicalTrials.gov, NCT02556424. Registered on 22 September 2015.

## Background

Any ocular inflammation may be complicated by inflammatory macular oedema. All cases of uveitis may potentially lead to macular oedema, with varying frequency according to location [1].

Epidemiological studies offer little information about blindness from uveitis, yet uveitis is the fifth leading cause of legal blindness in adults aged 20–65 years in the western world and is responsible for 10–15% of cases of total blindness in the United States [2]. Macular oedema is present in one-third of cases of uveitis in the Lardenoye series [1], and is the leading complication causing blindness in uveitis. It is responsible for 26.8% to 42% of acute visual loss [1, 3], comparable to the older Rothova series, which pointed to inflammatory macular oedema as being responsible for 29% of legal blindness and 41% of decreases in visual acuity [4].

The mechanisms of disruption to the blood–retinal barrier that cause inflammatory macular oedema are many and varied. Disruption to the blood–retinal barrier causes exudation of plasma proteins and lipids which have oncotic properties that result in retention of the liquid mainly in the extracellular space; this is the macular oedema. The disruption to homeostasis and retinal detoxification also causes macular cell death, which explains the absence of ad integrum recovery of visual acuity in cases of extended inflammatory macular oedema.

Depending on its severity, the macular oedema may remain asymptomatic, or it may cause macular degeneration of varying complexity—decreased near and far visual acuity, metamorphopsia, central scotoma, central phosphenes and micropsia. Symptoms of the inflammatory condition responsible for macular oedema may be present and mask the signs associated with macular oedema, hence the importance of systematic testing in uveitis.

There is no consensus on the therapeutic management of inflammatory macular oedema [5]. Similarly, there is no clear definition of “clinically significant” inflammatory macular oedema, as there is in diabetes, to provide a basis for comparison of the different studies.

Many hypotheses have been advanced to explain how various conditions can cause macular oedema. The inflammatory hypothesis is based on the increase in proinflammatory cytokines and in vascular endothelial growth factor expression which is central to the dynamics of inflammatory macular oedema. However, the mechanisms underlying inflammatory macular oedema are varied and may involve mechanical, toxic or inflammatory factors.

Oral steroids are better suited to the treatment of bilateral inflammatory attacks or when the use of a topical treatment is not possible. They are usually administered in the form of prednisone on account of its enhanced bioavailability compared to prednisolone.

Peri- and intraocular routes are used to limit the systemic side effects of anti-inflammatory steroids. Weijtens et al. showed that the intravitreal route enabled a maximum concentration of vitreous corticosteroid to be obtained, followed by the subconjunctival and peribulbar routes, for which the concentrations achieved were 120 and 13 times higher, respectively, than after oral administration [6–9].

The justification for the subconjunctival route is that the intravitreal corticosteroid concentration is 15 times higher after a subconjunctival injection of 2.5 mg dexamethasone 700 μg than after taking 50 mg of oral prednisone for several days [6]. Regarding peribulbar injections, some results provide evidence of intraocular penetration through the lamina rather than through the sclera, with the trans-scleral passage of molecules being disrupted by the choroidal blood flow as well as by the low permeability of the pigmented epithelial layer of the retina; this could be an argument in favour of subconjunctival injections instead of peribulbar or potentially sub-Tenon’s injections [6]. It is essential to remember that such periocular treatment (subconjunctival or peribulbar) is not a purely local treatment, to the extent that the blood corticosteroid concentration is found to be comparable to that of oral treatment.

**Fig. 1:**
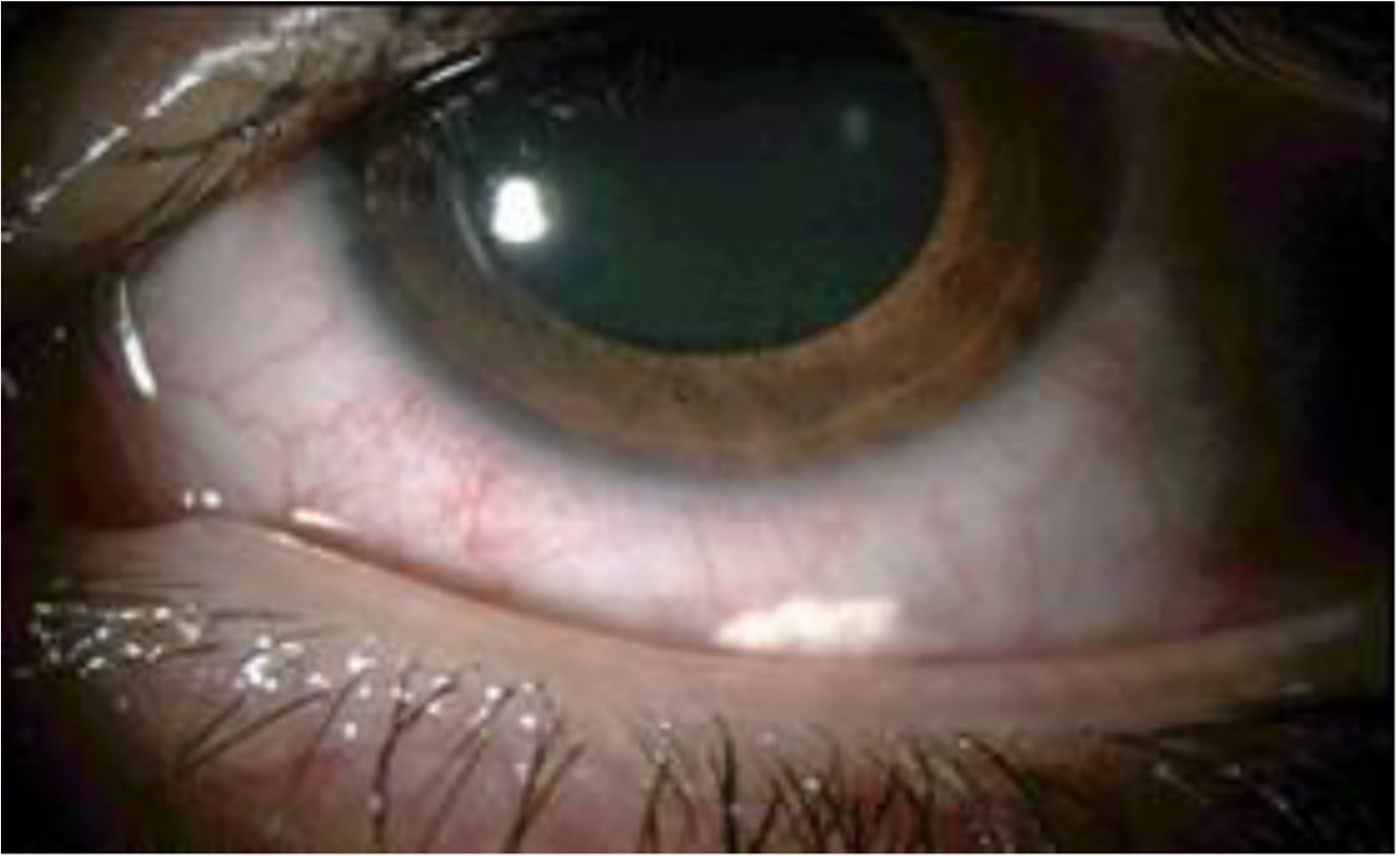
Subconjunctival triamcinolone crystals (from Turpin et al. [11])

In practical terms, subconjunctival injections are easily achievable in routine care. In case of complications, crystals can be easily removed under local anaesthesia in the operating room. Kalina et al. showed that removal of subconjunctival triamcinolone crystals is effective in normalising intraocular pressure [10] (Fig. 1).

The arrival on the market of the dexamethasone 700 μg intravitreal device has changed current practice. Its use is now authorized for first-line treatment of macular oedema secondary to occlusion of the central retinal vein and venous branches. It is also indicated for the treatment of noninfectious posterior uveitis and is being investigated for diabetic macular oedema.

Intravitreal injections require greater material, human and financial resources than subconjunctival injections since they must be performed in the operating room or in a dedicated room that meets specific criteria for strictly aseptic conditions.

Complications can occur and may be transient or permanent and may require medical or surgical treatment. The most common complication, subconjunctival haemorrhage, is trivial. In rare cases, endophthalmitis, elevated intraocular pressure (IOP) requiring medical or surgical treatment, damage to the lens causing a cataract, intravitreal haemorrhage, or retinal detachment may occur.

The ocular complications of corticosteroids are summarized in Additional file 1.

The efficacy and safety of triamcinolone in macular oedema, including inflammatory oedema, have already been studied [12–14]. For many years, the Department of Ophthalmology of CHU Nantes (University Hospital of Nantes), and other institutions, used subconjunctival triamcinolone injections. Since they are easy to perform extemporaneously on the day of consultation, they appear to be very effective in inflammatory macular oedema, both anatomically and functionally, with few complications. Unfortunately, there are currently no publications on these simple, effective and well-tolerated injections. Similarly, the sub-Tenon’s route is a possibility, but is more complex to execute.

Finally, the dexamethasone 700 μg implant has been authorized for the treatment of noninfectious intermediate and posterior uveitis, but there have been no studies to evaluate the efficacy and safety of the different peri- and intraocular strategies, including the treatment of inflammatory macular oedema.

Ozurdex® (i.e. dexamethasone 700 μg) consists of a biodegradable copolymer of glycolic acid and lactic acid and 700 μg dexamethasone which is gradually released into the eye [15, 16]. The clinical safety of Ozurdex® in patients with inflammation of the posterior segment of the eye presenting as noninfectious uveitis was assessed in a single, multicentre, masked, randomised study known as Huron [17]. The most frequently reported adverse reactions in the eye of patients who received Ozurdex® were conjunctival haemorrhage (30.3%), increased IOP (25.0%) and cataract (11.8%).

Pivotal studies and real-life studies have confirmed that the safety profile of Ozurdex® is good, with the same complications of cataract progression in the range of 29.8% [18] to 67.9% [19], closely related to the number of implants received, and an increase in IOP >10 mmHg from baseline reported in 15.4% to 27.7% of cases [19].

Furthermore, in real life, it has been shown that shorter interval retreatment is required because the drug is effective for less than 6 months, with a reported range that varies from 4 to 5.9 months [20, 21]. Indeed, drug release peaks at 2 months and there is then a steady decline that prolongs its effects for up to 6 months [16].

Our department was one of the first and only teams to perform a retrospective study of patients treated with Ozurdex® versus subconjunctival triamcinolone versus sub-Tenon’s triamcinolone [22]. This study of 88 patients demonstrated neither superiority nor any difference in the efficacy and safety of the three treatments.

Moreover, we must emphasize the potential impact on public health of a randomised prospective trial if subconjunctival injections of triamcinolone (a Kenacort retard® bulb costs €2.84 and the injection is performed in the physician’s office, with no additional overhead costs) were to prove at least as effective as injection of the dexamethasone 700 μg implant (Ozurdex® costs €962.65 and each injection must be performed in a dedicated room).

Our question is, how do intravitreal injections of a dexamethasone 700 μg implant and subconjunctival triamcinolone injections compare in terms of efficacy and safety? The arrival on the market of the dexamethasone 700 μg implant with authorisation for the treatment of posterior and intermediate uveitis tends to eliminate subconjunctival triamcinolone injections. However, these are simple, effective and well tolerated; they have the advantage that they are not delivered intraocularly, and they cost less.

## Methods/design

### Study design

**Fig. 2:**
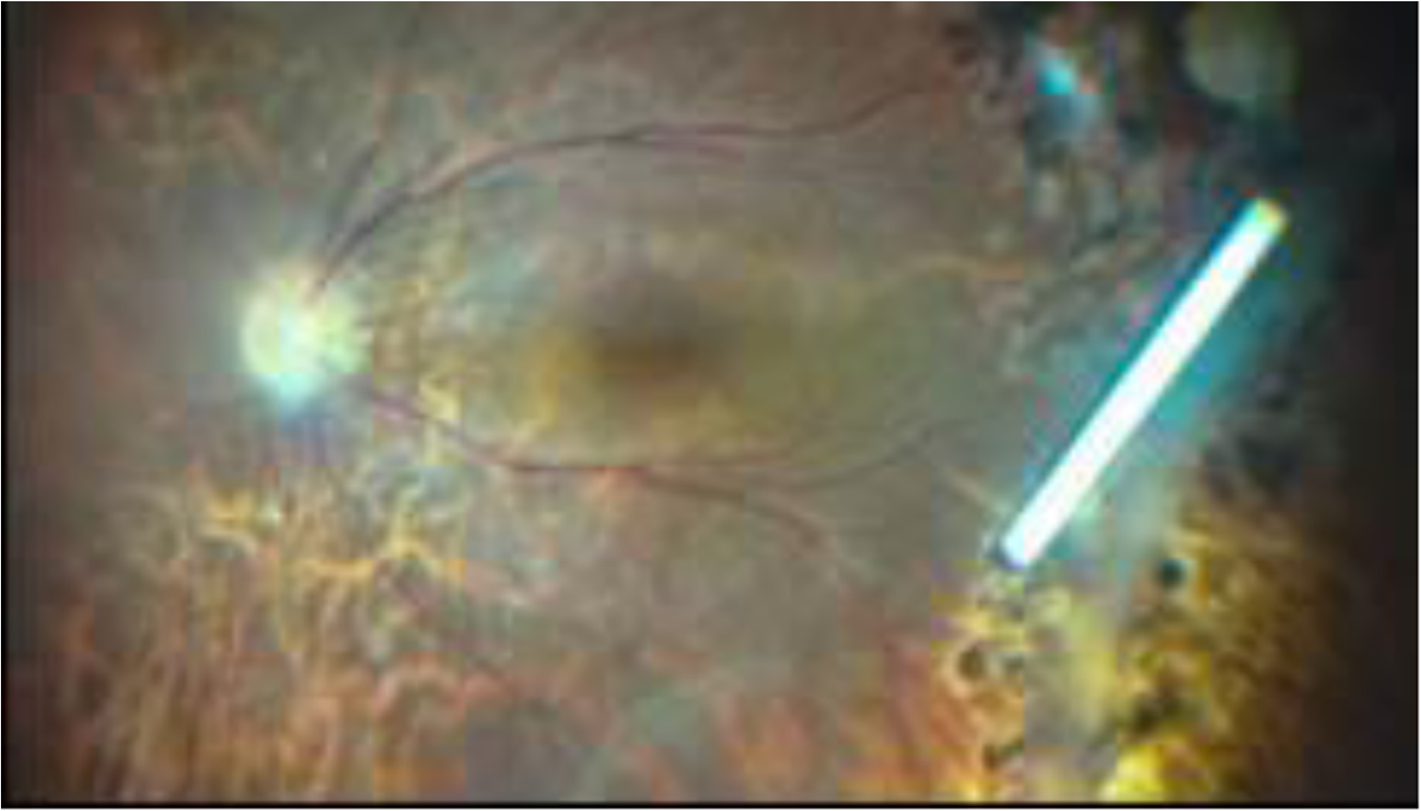
An intravitreal dexamethasone 700 μg implant (from Turpin et al. [11])

The TRIOZ clinical trial is an open-label, prospective, randomised study. For technical and ethical reasons, it is not possible to inject two products (drug versus placebo) in two different injections to maintain the masking; furthermore, the corticosteroids are visible to the investigator during control examinations (subconjunctival crystals, Fig. 1; intravitreal implant, Fig. 2). However, it was planned that visual acuity and central macular thickness (CMT) would be assessed by an ophthalmologist unacquainted with the trial who had not attended the patient’s surgery. The choice of these end points with a masked team allows the primary outcome and one of the secondary outcomes to be evaluated masked. No other assessments can be made on a masked basis, since the examination itself may reveal to the ophthalmologist which treatment the patient has received. Logistically, however, and especially due to the number of people in charge of clinical research in every centre, it was very difficult to create two teams, one masked and the other open, so the data have therefore been extracted on an open basis.

### Study population

#### Description of the population

Recruitment is planned over a period of 60 months in 14 French study centres: the Ophthalmology Departments in the University Hospitals of Nantes, Lille, Lyon, Tours, Brest, Paris (Pitié Salpêtrière), Paris (Kremlin Bicêtre), Paris (Fondation Rothschild), Bordeaux, Nancy, Grenoble, Nice, Montpellier and Dijon.

Inflammatory macular oedema is a common pathology found in each centre at the rate of 10 cases per centre per month, making these recruitment targets achievable.

#### Recruitment for the trial

Patients of both sexes, aged over 18 years with inflammatory macular oedema (meaning that they have a CMT >320 μm), will be recruited by the Ophthalmology Departments of the 14 French centres participating in the trial. For patients with bilateral asymmetric inflammatory macular oedema, the eye most affected will be treated. These patients should also be healthy and should not present a progressive disease. Patients who are HIV positive or who have hepatitis B or C virus, syphilis (TPHA-VDRL) or tuberculosis (Quantiferon) will not be included. Additional file 2 presents all the inclusion and exclusion criteria.

### Study schedule

**Fig. 3:**
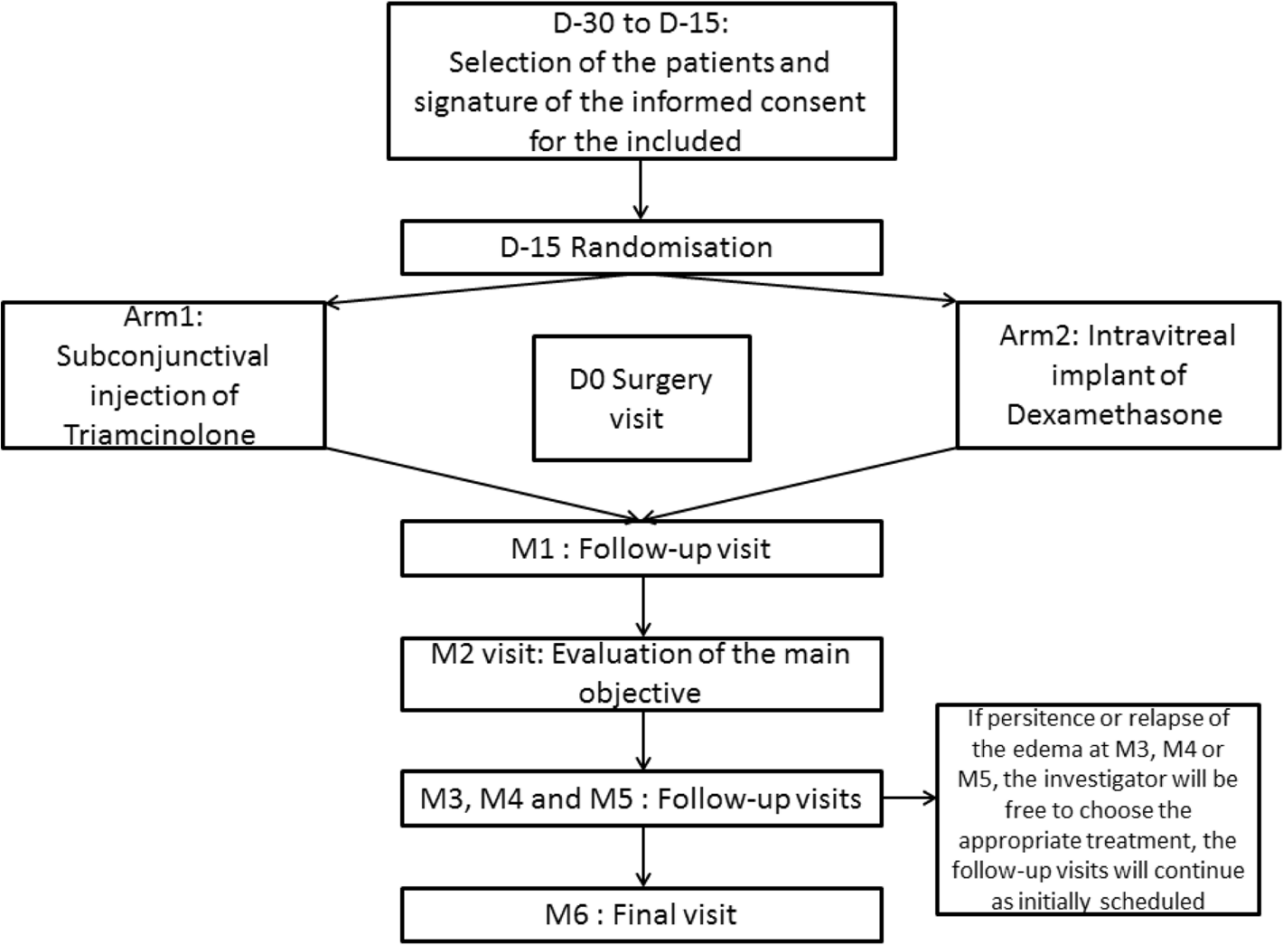
Flowchart: overview of the enrolment and follow-up of study participants. D day, M month

The plan for the study described in this section is presented in Fig. 3, and Additional file 3 shows the time points at which the assessments are to be made.

Before proceeding with any examination related to the research, the investigator obtains the patient’s freely given, informed consent, in writing.

At the inclusion visit, the investigating physician provides the patient with information on and answers any questions about the purpose of the research, the demands of the study, the foreseeable risks and the expected benefits. They also specify the patient’s rights when taking part in biomedical research and verify the eligibility criteria. The investigating physician then gives the patient a copy of the information form and consent form. Patients who do not consent to the trial will be treated according to standard care.

After the briefing, the patient has a reasonable period of reflection between receiving the information and consent forms and making a decision.

The investigative physician is responsible for obtaining the patient’s written informed consent. The consent form must be signed prior to any clinical or paraclinical examination required for the research. If the patient cannot read the information letter and informed consent form, their companion shall read their documents and countersign the consent.

The different copies of the information form and consent form are then distributed as follows: 1) the patient receives a copy of the information form, a signed consent form (see Additional file 4) and a patient card; and 2) the investigating physician keeps the original copy (even when the patient is moved for the duration of the research) in the investigator’s file.

The investigative physician will check the inclusion and exclusion criteria again at the inclusion visit and after signing the consent form, and will note the patient’s medical history and concomitant medications.

#### Screening visit

The following screening examinations will be conducted before the surgery visit (days (D)–30 to D0): 1) measurement of CMT using optical coherence tomography (OCT); 2) measurement of visual acuity (using the Early Treatment Diabetic Retinopathy Study (ETDRS) scale), a basic visual function parameter; 3) measurement of IOP, as hypertension is a major complication of peri- and intraocular corticosteroid injection; 4) examination of the anterior segment by slit lamp (SL) to quantify the flare and assess the clarity of the lens since the second most common complication of peri- and intraocular corticosteroids is development of a cataract; 5) examination of the posterior segment (fundus) and nonmydriatic fundus photography to assess vitreous haze; 6) examination of fluorescein angiography to detect vasculitis, an associated papillitis; 7) automated quantitative and objective measurement of the flare when available using a laser flare meter (LFM), which enables a more accurate assessment of the status and previous inflammatory condition of the blood–aqueous barrier (reference value); 8) measurement of blood pressure, as systemic corticosteroids can induce hypertension and the pathways around the eyes especially mean that treatment is not purely local (exclusion criterion); and 9) blood tests for fasting plasma glucose and glycohaemoglobin (because systemic corticosteroids can induce diabetes and, more importantly, the pathways around the eyes mean that treatment is not purely local; and exclusion criterion). In addition, a pregnancy test (beta human chorionic gonadotropin) will be conducted for women of childbearing age; pregnant women are excluded from the protocol even though peri- and intraocular corticosteroids are permitted during pregnancy. Finally, a serology test will be made for HIV, hepatitis B and C virus, TPHA-VDRL and Quantiferon if the status is unknown to eliminate macular oedema due to infection and systemic infectious diseases at risk of aggravation by treatment with corticosteroids, with the exception of obvious postoperative inflammation.

#### Treatment visit

At the beginning of the treatment visit (D0; injection of the product), the patient will answer their first EuroQol five dimensions (EQ-5D) questionnaire.

Before either the dexamethasone 700 μg implant or triamcinolone is injected, anaesthetic and antiseptic eye drops will be given according to the centre’s practices. Analgesics are permitted. Initiation of hypotensive eye drops for curative or preventive purposes is allowed. The investigator must report this in the case report form (CRF). According to the exclusion criteria, general anti-inflammatory treatments or systemic immunosuppressive or immunomodulatory treatments at unstable doses are not authorised during the trial. From D0, acetazolamide cannot be continued or introduced later.

For the subconjunctival triamcinolone injection (Kenacort retard®), the patient is positioned comfortably. The injection is performed after the instillation of anaesthetic drops. Triamcinolone injections are carried out using a 25-gauge needle. A volume of 0.4 ml is injected under the inferior bulbar conjunctiva so that the eyelid covers the visible crystals without discomfort.

**Fig. 4:**
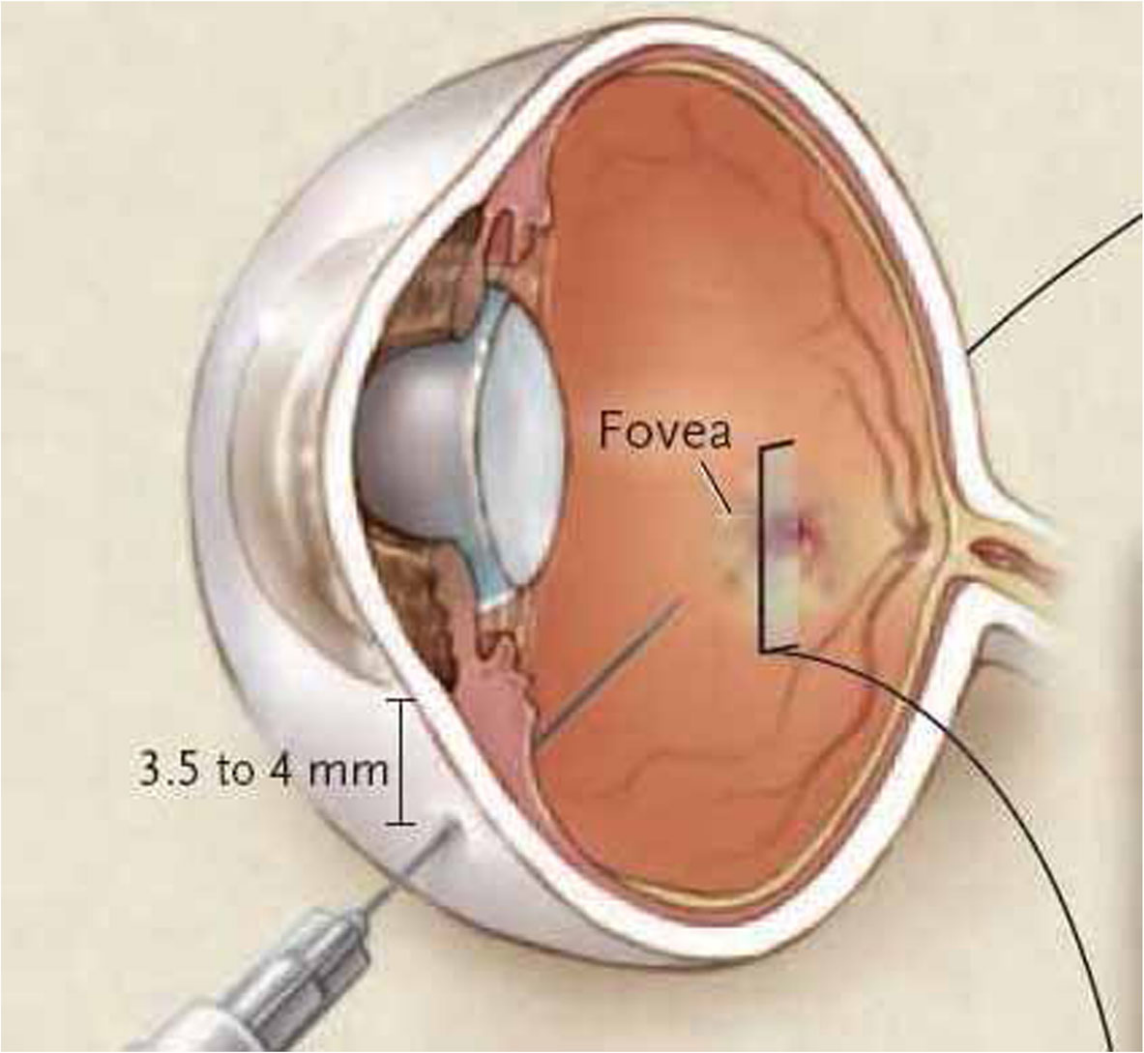
Intravitreal injection (from Turpin et al. [11])

For Ozurdex®, the injection is performed in the supine or semi-seated position. The eye is numbed with the instillation of anaesthetic eye drops. The eyelids and ocular surface are disinfected with antiseptic to reduce the risk of infection. The face is covered with a sterile drape and a sterile eyelid retractor is positioned. The injection device for the dexamethasone 700 μg implant is sterile and ready to use; it is inserted through the sclera 3.5 mm (pseudophakic eye) or 4 mm (phakic eye) from the lamina after moving the conjunctiva a few millimetres (Fig. 4). Following the injection, antibiotic eyedrops are instilled. The treated eye remains painless in the vast majority of cases. A spot may appear in the visual field, corresponding to the presence of the implant in the vitreous cavity (Fig. 2).

After treatment, the investigator will give the patient a tray with a 10-cm slider for scoring the injection pain from 0 (no pain) to 10 (unbearable pain). The pointer will provide physicians with a measurement of pain in centimetres.

The investigator will then ask the patient to rate the injection, choosing between “tolerable”, “uncomfortable” and “very unpleasant”.

The physician will record the patient’s concomitant medications and any adverse events (AEs) and serious adverse events (SAEs). All such events will be reported to the person responsible for pharmacovigilance in clinical trials at CHU Nantes.

Please note that as the treatment takes place at hospital there is no need to monitor adherence.

#### Follow-up visits

The monthly follow-up visits (M1–M6) allow for early detection of efficacy and complications (including ocular hypertonia) beginning within approximately 1 month [12, 23]. In addition, they provide reassurance for the patient and reduce protocol deviations and patients lost to follow-up.

The investigating physician will record any AEs/SAEs and any concomitant medications.

They will include the following in their examinations of the patient during visits M1, M2, M3, M4, M5 and M6 (visits M1 and M4 do not form part of the usual care): 1) measurement of CMT (by OCT); 2) measurement of visual acuity (using the ETDRS scale); 3) measurement of IOP; 4) examination of the anterior segment (by SL) for flare and crystalline lens clarity; 5) examination of the posterior segment (fundus) and nonmydriatic fundus photography for posterior vitreous haze; and 6) automated measurement of the flare (by LFM), if available. In addition, the patient will complete the EQ-5D questionnaire.

At M3 and M6, the patient’s blood pressure will be taken. A fluorescein angiograph is conducted at M2 and M6 if anomalies were detected at the inclusion visit. In addition, a blood sample will be taken at M6 to evaluate haemoglycaemia and fasting plasma glucose.

In cases of inadequate response or relapse between the M3 visit and the M6 visit, the investigator will be free to choose the appropriate treatment on the day that the inadequate response/relapse is detected, or later (± 30 days), at their discretion; follow-up visits will continue according to the initial schedule, i.e. monthly visits.

The research may be discontinued as a result of: 1) the patient withdrawing their consent; 2) necessity as decided by the investigator in case of SAEs that prevent the patient from continuing with the protocol; or 3) a decision by the authorities or the suspension/withdrawal of the drugs from the market.

In case of premature discontinuation from the study, the patient will be referred immediately to the investigator for a consultation to provide care for their disease. The premature end of study visit will be completed during this consultation.

### Objectives and statistics

#### Objectives

The main objective of our study is to evaluate the efficacy of subconjunctival injection of triamcinolone in reducing CMT measured by OCT, the most objective, relevant and noninvasive criterion, compared with intravitreal injection of a dexamethasone 700 μg implant which has been approved for this indication, between the inclusion visit and 2 months after treatment (M2 visit).

The timeframe was chosen based on data from the Huron study of Ozurdex® [17, 24, 25] and data from our retrospective study on triamcinolone in the department at CHU Nantes [22].

The secondary objectives are: 1) evaluation of the experience of the injection; 2) evaluation of the effectiveness of the injection studied at each visit, measured by the gain in visual acuity (by the EDTRS scale; a key parameter of visual function), the reduction in flare and vitreous haze which are quantifiable ocular inflammatory parameters measured using an SL and LFM in centres equipped with this technology, the CMT measured using OCT (enabling evaluation of the duration of action of a subconjunctival triamcinolone injection compared with an intravitreal injection of a dexamethasone 700 μg implant; the duration of action of the injection is determined by the reappearance of oedema), the local and general tolerance of the two methods (collecting details of all AEs/SAEs) and the patients’ quality of life; and 3) economic evaluation of the efficiency (cost–utility analysis) of the subconjunctival triamcinolone injection compared with intravitreal injection of a dexamethasone implant from a societal perspective and over a 6-month period.

#### Primary outcome

The primary outcome is the difference in CMT in the treated eye, measured using spectral-domain OCT in both groups between selection and M2.

The micrometric CMT was converted into a logarithmic CMT (logSD-OCT) for statistical analysis, considering that the normal CMT was 250 μm.

The formula used in the trial is: logarithmic CMT = log10 (micrometric CMT/250).

Use of a logarithmic scale for analysing changes in CMT gives a more normal distribution for CMTs that coincides with the distribution of logarithmic visual acuity. Some studies have shown that the logarithmic transformation of the CMT provides a better picture of visual acuity [26, 27].

Our CMT results were expressed in microns and logSD-OCT so they could be compared with those in the literature.

A retrospective study in the department at CHU de Nantes on the effectiveness of triamcinolone injections in reducing CMT converted into logSD-OCT obtained the following results: between M0 and M1, −0.12logSD-OCT (*p* < 0.001) and between M0 and M3, −0.09logSD-OCT (*p* = 0.002), introducing the value of M2 as the main criterion.

#### Secondary outcomes

Secondary outcomes of the study are: 1) the scoring of “the moment” of injection on the day of injection (tolerable, uncomfortable, very unpleasant) and the rating on a visual analogue scale (from 0 cm = no pain to 10 cm = extreme pain); 2) visual acuity (ETDRS scale) at every visit to determine the gain between the inclusion visit and the follow-up visits (the mean scores for each arm will be compared at each follow-up visit); 3) the flare (using SL and LFM if available) at every visit to determine the reduction between the inclusion visit and the follow-up visit (the mean scores for each arm will be compared at each follow-up visit); 4) the vitreous haze at every visit to determine the reduction between the inclusion visit and the follow-up visit (the mean scores for each arm will be compared at each follow-up visit); 5) the thickness of the central macula of the treated eye to determine the duration of action at every visit (as stated in the objectives, the duration of action of the injection is determined by the reappearance of oedema; the mean scores for each arm will be compared at each follow-up visit); 6) AEs/SAEs including intermittent ocular hypertension, cataract, endophthalmitis, glycaemic and blood pressure imbalances at every visit; and 7) EQ-5D questionnaire on patient quality of life at every visit.

#### Efficiency outcomes

The incremental cost-effectiveness ratio (ICER; cost per quality-adjusted life-year (QALY)) will be calculated for the comparison between subconjunctival triamcinolone injection and intravitreal injection of a dexamethasone implant from a societal perspective and over a 6-month time period.

#### Measures used to determine the outcomes

OCT is used to accurately visualise the different layers of the retina, including the macula, using an infrared laser. This is a contactless examination which is noninvasive, painless and brief.

An LFM is a device used to measure the protein concentration in the anterior chamber using a helium–neon laser. This is a contactless examination that is non-invasive, painless and brief.

All the ophthalmic examinations performed at inclusion and during follow-up are common practice in ophthalmology. The visual acuity, IOP, examination using the SL and fundus examination form the basis of all clinical ophthalmic examinations. OCT is the test of choice for characterising and quantifying macular oedema, irrespective of type. This testing is contactless and is noninvasive, painless and brief. The values considered to be normal are: visual acuity, ETDRS 100; IOP, 12–21 mmHg; LFM, no proteinic flare or cellular Tyndall; fundus, no cellular Tyndall or vitreous haze; macula as described, normally LFM <10 ph/ms, CMT <300 μm and >250 μm.

The patient will be given the EQ-5D questionnaire validated in France [28, 29] at each visit to measure their quality of life. The EQ-5D consists of a questionnaire and a visual analogue scale. The questionnaire focuses on five areas: mobility, personal autonomy, current activities, pain/discomfort and anxiety/depression. There are three possible answers for each of these dimensions (EQ-5D-3 L), thus allowing for 243 health states. QALYs will be calculated for each arm using area under the curve methodology and the weighting coefficients available in France for the EQ-5D-3 L [29, 30].

#### Statistical methods

The data will be reviewed at the end of the study, prior to statistical analysis. The aim will be to review the progress of the study, identify potential problems and classify any minor or major deviations.

The variables measured at baseline will be described for all patients in both groups in terms of numbers and percentages for each category for categorical variables and minimum, maximum, average, standard deviation and quartile values for quantitative variables.

The primary endpoint is the difference in CMT in the treated eye between D0 and M2. CMT measurements will be converted into logarithmic CMT: logarithmic CMT = log10(CMT/250).

The main objective is to demonstrate the noninferiority of the group with subconjunctival triamcinolone injection compared with the group with intravitreal injection of a dexamethasone implant. The noninferiority margin was set at 0.06 (equivalent to CMT = 287). To demonstrate the noninferiority of the triamcinolone group versus the dexamethasone group, the 95% bilateral confidence interval (CI) of the difference between the two groups (dexamethasone – triamcinolone) will be estimated using a mixed linear regression model. This model will reflect the stratification factor of randomisation to the centre (the centre will be considered as a random effect) and will be adjusted according to the measurement at D0. The estimated upper boundary of the CI will be compared to the predefined noninferiority margin. If the upper boundary is less than 0.06, the noninferiority of the triamcinolone group as compared with the dexamethasone group will be demonstrated. For missing data, the worst observed value will be imputed for triamcinolone patients and the best value for dexamethasone patients. A sensitivity analysis will be performed with multiple imputation.

For secondary end points, the mean visual analogue scale score evaluating the moment of injection will be compared between groups using a linear mixed model; the score for the moment (tolerable, uncomfortable or very unpleasant) will be compared using the Mantel–Haenszel stratified Chi-squared test. A linear mixed model will be used to compare the change in ETDRS from D0 to 6 months between the groups. SL and LFM between D0 and 2 months will be compared between the groups using nonparametric Van Elteren tests (semiquantitative outcome). Duration of injection efficacy (duration is determined by the reappearance of oedema) will be compared between the groups using the Van Elteren test (semiquantitative outcome). Descriptions of AEs/SAEs will be reported in the two groups. Comparisons between the groups will be performed using Chi-squared or Fisher tests for intermittent ocular hypertension, cataract, endophthalmitis, glycaemic and blood pressure imbalances in both arms. There will be no imputation for missing data for these secondary end points.

For the economic assessment, mean costs and their corresponding 95% CIs will be presented. The ICERs will be estimated along with their corresponding acceptability curves, i.e. the curves indicating the probability that an intervention is cost effective conditional on society’s willingness to pay for an additional unit of effectiveness (i.e. an additional QALY gained) and considering the sampling uncertainty around the estimated ICERs.

As costs and ICERs are not normally distributed, the 95% CI and the cost-effectiveness acceptability curves will be determined using the nonparametric bootstrap resampling technique.

Sensitivity analyses will be performed for all end points, adjusted for the duration of macular oedema. All statistical tests will be bilateral. For secondary endpoints, a *P* value less than 0.05 will be considered statistically significant. Analyses will be performed using SAS statistical software (SAS Institute Inc., Cary, NC, USA).

As this is a noninferiority study, the analyses will be carried out on the intent-to-treat population and on the per-protocol population. The intent-to-treat population consists of all randomised patients in the study. The per-protocol population includes the most compliant patients, based on compliance with the inclusion and exclusion criteria, absence of major deviations from the protocol and availability of the main criterion.

#### Sample size

This TRIOZ trial aims to demonstrate the noninferiority of subconjunctival triamcinolone injections compared to intravitreal dexamethasone implants. Noninferiority will be evaluated in terms of the difference in macular thickness in the treated eye between D0 and M2. The deadline of M2 was chosen based on the plan for the Huron study on dexamethasone (NCT000333814) and a retrospective study on triamcinolone at CHU Nantes. Preliminary data observed retrospectively in Nantes between 2011 and 2013 in 25 patients who received triamcinolone injections showed a decrease of 0.12 ± 0.12 log OCT at M1 (D0, 0.27 ± 0.11; M1, 0.15 ± 0.08) and of 0.09 at M3 (0.18 ± 0.11). The difference in macular thickness between D0 and M2 is assumed to be the same in the two groups and the common standard deviation is set at 0.12. The noninferiority margin was set at 0.06, the power at 80% and the type I error rate at 2.5%. Based on these assumptions, 128 patients are needed to demonstrate the noninferiority of triamcinolone compared to dexamethasone. A maximum rate of 10% for missing data is taken into consideration for M2 and 142 patients, or 71 patients per group, will therefore be randomised in the study.

#### Randomisation

Randomisation will be conducted openly and stratified by centre. It will be performed according to a 1:1 ratio and balanced by blocks. The random numbers will be generated by computer. Subjects are randomised into blocks as the allocation progresses, a block being a subgroup of predetermined size within which there is a random allocation of patients. The software used for the randomisation is SAS version 9.4. The randomisation key is known only to the biostatistician and the data managers to make it impossible for the investigator to assign a particular treatment.

As mentioned above, for logistical reasons the trial is now open-label. As Karanicolas et al. pointed out in their article from 2010, the study should have been masked to the biostatisticians until analyses were performed to reduce the study bias [31].

Logistically, as Ozurdex® requires an operating room, randomisation will be performed 15 days prior to the date of surgery. After confirming the inclusion/exclusion criteria on the electronic CRF, the investigator will perform the randomisation without the patient present; the patient will only know to what treatment they have been assigned at the treatment visit.

Upon activation, each study centre will receive two batches, each containing triamcinolone and Ozurdex® for the treatment of the first two patients. Based on the randomisation, the batch used will be replaced by the pharmacy of CHU Nantes. The treatments will be kept at the pharmacy in each study centre for issue on the prescription of the investigator during the treatment visit.

### Adverse event management

There are no pharmacokinetic data in the literature for triamcinolone administered subconjunctivally. The clinical experience of the various centres performing these injections shows a duration of action of approximately 3 to 4 months for 0.3 and 0.4 ml, respectively. The duration of action of an intravitreal injection of a dexamethasone 700 μg implant is approximately 3 to 6 months.

Inflammatory macular oedema, a chronic and recurrent pathology, requires regular ophthalmological monitoring at least every 6 months, regardless of the type of treatment administered.

The most frequently occurring adverse reactions identified based on the summary of product characteristics for Ozurdex® and the experience of the University Hospital of Nantes for triamcinolone are: 1) corticosteroid-induced hypertension (eye tone will be monitored monthly until the effectiveness of any injected corticosteroid has been exhausted and at longer intervals thereafter based on blood pressure control); and 2) cataract (the visual acuity and appearance of the lens will be monitored using an SL during the usual follow-up consultations for inflammatory macular oedema until cataract surgery is performed).

All the AEs encountered that are observed by the investigator or reported by the subject during the study, whether or not they are expected (see the summary of product characteristics for Ozurdex®), should be documented in the AE section of the CRF.

#### SAE reporting

All SAEs, whether expected or unexpected, require the completion of an SAE report. The investigator must ensure that the information entered in this report is accurate and clear. The SAE should be reported immediately (within 24 h of being highlighted by the investigator) to the sponsor. After receiving an unexpected SAE report, the sponsor notifies the authorities. Once a year, the sponsor prepares an annual safety report.

Furthermore, a Data and Safety Monitoring Committee (DSMC) has been set up. This is a consultative committee responsible for reviewing the safety of a study on behalf of the sponsor and the coordinator/principal investigator of the study. Members of the committee who are competent in the field of clinical trials (pathology and methodology) are not involved in the study.

The DSMC receives the annual safety reports and is a point of referral for pharmacovigilance if a suspected unexpected serious adverse reaction or an SAE poses particular analytical difficulty or if a doubt arises in the study about the risk/benefit.

In the event of early termination of the study by a decision of the DSMC or the study sponsor, the regulatory authorities and the Ethical Review Board will be informed by post within a maximum of 15 days.

In any event, written confirmation will be sent to the coordinating investigator for the study (specifying the reasons for early termination) and to the principal investigator of each centre, if applicable. All patients in the study will be informed and will be required to complete their early discharge visit.

### Ethical, regulatory and dissemination aspects

The clinical study will be conducted in accordance with the relevant versions of the French Public Health Code, national and international Good Clinical Practice guidelines, and the Declaration of Helsinki, each in the applicable version.

In compliance with French law, the study protocol was submitted to the French regulatory authority (ANSM) and was approved on 31 August 2015.

This clinical study was submitted to and approved by the Ethical Review Board of Angers (Comité de Protection des Personnes – CPP OUEST II - Angers) on 24 August, 2015 (see Additional file 4 for French informed consent). Requests for substantial modifications should be addressed by the sponsor for approval or notification to ANSM and/or the Ethical Review Board concerned in compliance with Law 2004–806 of 9 August 2004 and its implementing decrees.

The clinical protocol has been writeen according to Spirit check-list (see Additionnal file 5). The amended protocol should be a dated, updated version. If necessary, the information form and consent form should be amended.

The updated protocol is at version 10 on 7 July 2018.

All the submissions/declarations were made by the Sponsor Department at CHU Nantes which manages the quality of the data collected. The data collected during the study will be processed electronically in accordance with the requirements of the CNIL, the French Data Protection Authority (in compliance with the French Reference Methodology MR001).

As required, the sponsor has provided an insurance policy to cover the financial consequences of its civil liability in accordance with the regulations.

It has been possible to carry out the protocol and the trial thanks to an Executive Committee which includes a Scientific Committee and a Steering Committee. The Scientific Committee was created by M. Weber and its membership comprises external experts in this pathology, biostatisticians and methodologists, the medical economist and the project manager of the clinical investigation centre (CIC1413). It is coordinated by Dr. Couret. The Steering Committee is composed of the members of the Scientific Committee, except the external experts, and with the addition of the data management team, the nurse study coordinator from the Ophthalmology Department of CHU Nantes who coordinates assistance for patient inclusion in the other centres, and the monitoring Clinical Research Assistant (CRA). The sponsor project manager coordinates this committee and drafts the “TRIOZ newsletter” which provides, among other things, the latest news on patient inclusion, amendments to the protocol, and so forth.

An inspection or audit may take place as part of this study, performed by the sponsor and/or by the regulatory authorities. Inspectors will check the documents, logistics, records and any other resources that the authorities consider to be associated with the clinical trial and that may be located at the trial site itself.

The trial results will be published in international ophthalmological, medical and scientific journals and presented at national and international conferences. The investigators, who will share the entirety of the final trial dataset, will follow the rules and guidelines of the International Committee for Medical Journal Editors (ICMJE) [32]. In practice, the Scientific Committee will be among the authors of the publication, as will the investigators who have included the most patients in the trial. The trial sponsor and the French Ministry of Health, which provided the grant, must be cited in the publication.

## Discussion

Demonstration of the efficacy and safety of subconjunctival triamcinolone injections will enable their continued use at a time when the marketing authorisation for an intravitreal device requires the intraocular delivery of a unique, expensive compound, exposing the patient to a rare risk of endophthalmitis and retinal detachment, while the relative efficacy and safety of these approaches have never been compared.

The European Union has established a tight safety net. No medicinal product may be marketed in a member state unless the competent authorities of that state have issued a marketing authorisation [33]. Recently, a control framework for medically justified off-label prescriptions has been implemented in France. Act no. 2011–2012 of 29 December 2011 reinforcing the safety of medicines and health products [34] addresses the ambitious goal of controlling off-label drug use. The Act explicitly recognizes the right of physicians to prescribe drugs off-label for use by an individual patient, under their direct personal responsibility and in the patient’s interest. It also introduces a second, unique derogating provision into French law pursuant to Article L.5121-12-1 of the French Public Health Code, a regulatory process called “Temporary Recommendations for Use” (Recommandations Temporaires d’Utilisation (RTUs)) [35].

The French health authorities have amended the RTU framework in order to authorise the reimbursement of a drug used off-label, despite the existence of licensed therapeutic alternatives, because of the burden of licensed drugs on the health care system. To date, bevacizumab (Avastin®)/ranibizumab (Lucentis®) have provided an illustration of this “economic RTU”. Both drugs are antivascular endothelial growth factor agents and are made by the same parent company but only one, Lucentis®, has marketing authorisation for age-related macular degeneration (AMD). The other significant difference is the striking disparity in the cost of the two drugs. The new price of Avastin® is now €100 including VAT, compared to €10 initially; a monthly injection of Lucentis® is €738. Avastin® is registered for the treatment of systemic cancer but is used off-label to treat AMD. One study published in 2012 using data from Medicare 2010 surveyed physicians on why they chose Avastin® versus Lucentis®. Cost was reported as the primary factor for choosing bevacizumab (70%) [36]. In France, Avastin® has been indicated for the treatment of the neovascular form of AMD since 2015 under the economic RTU.

We hope, therefore, that if this clinical trial proves the efficiency of subconjunctival triamcinolone injections the French Health Authorities will authorise the reimbursement of this drug.

## Trial status

This trial is still ongoing; patient inclusion is not yet complete. The updated protocol is at version 10 on 7 July 2018. The first patient was included on 13 January 2016. Recruitment by the investigating centres is planned to continue until 13 October 2020 and the study period will end in March 2021.

## Supplementary information

**Additional file 1.** Major ocular complications of steroids by route of administration (from Turpin et al. [11]).

**Additional file 2.** Inclusion and exclusion criteria.

**Additional file 3.** TRIOZ study schedule.

**Additional file 4.** TRIOZ informed consent.

**Additional file 5.** SPIRIT 2013 checklist: recommended items to address in a clinical trial protocol and related documents.

## Data Availability

Data sharing is not applicable to this paper as no datasets were generated or analysed during the current study. The data from the completed trial will not be shared and will only be transmitted to the sponsor. Data collected during the test may be processed electronically, in accordance with the requirements of the CNIL (compliance with reference methodology MR001).

## Abbreviations

AE: Adverse event
AMD: Age-related macular degeneration
CI: Confidence interval
CMT: Central macular thickness
CRF: Case report form
DSMC: Data and Safety Monitoring Committee
EQ-5D: EuroQol five dimensions
ETDRS: Early Treatment Diabetic Retinopathy Study
ICER: Incremental cost effectiveness
IOP: Intraocular pressure
LFM: Laser flare meter
OCT: Optical coherence tomography
QALY: Quality-adjusted life year
SAE: Serious adverse event
SL: Slit lamp
