# Supplementary material for: Comparison of two techniques used in routine care for the treatment of inflammatory macular oedema, subconjunctival triamcinolone injection and intravitreal dexamethasone implant: medical and economic importance of this randomized controlled trial
Source: Trials. 2020 Feb 10;21:159. doi: 10.1186/s13063-020-4066-0 (PMC7011383; doi:10.1186/s13063-020-4066-0)
Supplement: Supplementary file 1 — Additional file 1. Major ocular complications of steroids by route of administration (from Turpin et al. [11]). [file 13063_2020_4066_MOESM1_ESM.doc]

| **Regardless of the route** | **Cataract,**  **Hypertension and glaucoma,**  **Recurrence of herpes infections,**  **Allergy to the excipient.** |
| --- | --- |
| **Topical** | Blurred vision,  Superficial punctate keratitis,  Delayed healing,  Superinfection,  Mydriasis, ptosis, rare problems of accommodation. |
| **Periocular (peri-bulbar > sub-Tenon of Nozik > sub-conjunctival)** | Occlusion of the central retinal artery.  Ptosis,  Fibrosis of oculomotor muscles,  Perforating trauma to the sclera or optic nerve. |
| **Intravitreal** | Endophthalmitis (0.1 to 0.5%) [34, 35],  Retinal detachment (1.8%) [36],  Retinal necrosis. |
| **Systemic** | Pseudotumor, exophthalmos,  Central serous chorioretinopathy. |
