# Supplementary material for: Comparison of two techniques used in routine care for the treatment of inflammatory macular oedema, subconjunctival triamcinolone injection and intravitreal dexamethasone implant: medical and economic importance of this randomized controlled trial
Source: Trials. 2020 Feb 10;21:159. doi: 10.1186/s13063-020-4066-0 (PMC7011383; doi:10.1186/s13063-020-4066-0)
Supplement: Supplementary file 2 — Additional file 2. Inclusion and exclusion criteria. [file 13063_2020_4066_MOESM2_ESM.doc]

| **Inclusion Criteria** | **Exclusion Criteria** |
| --- | --- |
| Patient :   - Male or female (under effective contraception if not menopausal) over 18 years - Affiliated with social security - Able to understand and follow the instructions of the study - Having signed an informed consent - Having a central macular thickness greater than 320μm (Spectral Domain, 270μm Time Domain). - With a unilateral or bilateral asymmetric inflammatory macular edema (in the case of a bilateral inflammatory macular edema, the eye most affected will be treated) | Patient :   - with infectious uveitis - HIV positive or affected by the hepatitis B or C virus (HBV HCV), syphilis (TPHA-VDRL) or tuberculosis (Quantiferon) - Receiving an unbalanced anti-inflammatory (recent modification <1 month) or general immunosuppressive therapy (recent modification <3 month) - With a personal history of glaucoma and/or ocular hypertension in the studied eye (intraocular pressure (IOP)> 25 mmHg without anti-glaucoma medication or > 21 mmHg in an antiglaucoma combination therapy) and/or a history of cortisone hypertension not controlled by an antiglaucoma combination therapy. - With uncontrolled diabetes (HbA1c>8%) or unbalanced hypertension (SBP> 160 mmHg and/or DBP> 100mmHg) - With an edematous diabetic maculopathy - Who had received triamcinolone subconjunctivally or sub-Tenon 3 months before randomization, or 6 months prior to the randomization of 700μg of dexamethasone intravitreally - With an history of ocular herpes infection, central serous chorioretinopathy - With an uncontrolled systemic disease. - Pregnant woman, or likely to become pregnant, or nursing - Participating in another clinical trial - Under guardianship |
