# Supplementary material for: Comparison of two techniques used in routine care for the treatment of inflammatory macular oedema, subconjunctival triamcinolone injection and intravitreal dexamethasone implant: medical and economic importance of this randomized controlled trial
Source: Trials. 2020 Feb 10;21:159. doi: 10.1186/s13063-020-4066-0 (PMC7011383; doi:10.1186/s13063-020-4066-0)
Supplement: Supplementary file 3 — Additional file 3. TRIOZ study schedule. [file 13063_2020_4066_MOESM3_ESM.doc]

|  | **D-30 to D0**  **Screening visit** | **D0**  **Treatment visit** | **M1**  **30 days from**  **D0 ± 7** | **M2**  **60 days from**  **D0 ± 7** | **M3**  **90 days from**  **D0 ± 7** | **M4**  **120 days from**  **D0 ± 7** | **M5**  **150 days from**  **D0 ± 7** | **M6**  **180 days from**  **D0 ±7** |
| --- | --- | --- | --- | --- | --- | --- | --- | --- |
| **Checking the inclusion/exclusion criteria** | X | X |  |  |  |  |  |  |
| **Informed consent** | X |  |  |  |  |  |  |  |
| **Medical history** | X |  |  |  |  |  |  |  |
| **Blood pressure** | X |  |  |  | X |  |  | X |
| **Measurement of central macular thickness (OCT)** | X |  | X* | X | X | X* | X | X |
| **Measurement of visual acuity (EDTRS)** | X |  | X* | X | X | X* | X | X |
| **IOP** | X |  | X | X | X | X | X | X |
| **Check if :**  **Hypotensive eye drops**  **Concomitant medication** | X  X | X  X | X  X | X  X | X  X | X  X | X  X | X  X |
| **Fundus exam** | X |  | X | X | X | X | X | X |
| **Examination of the anterior segment (SL)** | X |  | X | X | X | X | X | X |
| **Non-mydriatic fundus** | X |  | X* | X | X | X* | X | X |
| **Fluorescein angiography (FA - if an abnormality other than Macular Edema is detected)** | X |  |  | as decided by the investigator |  |  |  | as decided by the investigator |
| **Automated measurement of the Flare (if available)** | X |  | X | X | X | X | X | X |
| **Quality of life questionnaire EQ-5D** | X |  |  | X |  | X |  | X |
| **Pregnancy test** | X* |  |  |  |  |  |  |  |
| **FPG** | X |  |  |  |  |  |  | X |
| **Glycated hemoglobin** | X* |  |  |  |  |  |  | X* |
| **Any examination/analysis allowing, in case of doubt, to eliminate macular oedemas of infectious origin and to eliminate systemic infectious pathologies at risk of aggravation by corticosteroid treatment** | X |  |  |  |  |  |  |  |
| **Randomization** |  | X |  |  |  |  |  |  |
| **Treatment of oedema** |  | X |  |  |  |  |  |  |
| **Note if Relapse** |  |  |  |  | X | X | X | X |
| **Retreating at the choice of the investigator** |  |  |  |  | X | X | X | X |
| **VAS pain + moment of injection scale** |  | X |  |  |  |  |  |  |
| **Adverse events** |  | X | X | X | X | X | X | X |

*Act in addition to usual practice
