# Supplementary material for: Comparison of two techniques used in routine care for the treatment of inflammatory macular oedema, subconjunctival triamcinolone injection and intravitreal dexamethasone implant: medical and economic importance of this randomized controlled trial
Source: Trials. 2020 Feb 10;21:159. doi: 10.1186/s13063-020-4066-0 (PMC7011383; doi:10.1186/s13063-020-4066-0)
Supplement: Supplementary file 4 — Additional file 4. TRIOZ informed consent. [file 13063_2020_4066_MOESM4_ESM.pdf]

CENTRE HOSPITALIER UNIVERSITAIRE DE NANTES

Note d'information pour la participation à la recherche

« Comparaison de l'efficacité et de la tolérance d'une injection sous-conjonctivale de triamcinolone et d'un implant intravitréen de dexaméthasone sur l'œdème maculaire inflammatoire »

Titre abrégé : « TRIOZ »

Promoteur : CHU de Nantes

N° EudraCT : 2015-000468-33

Madame, Mademoiselle, Monsieur,

Dans le cadre de la prise en charge de votre maladie, l'œdème maculaire inflammatoire, nous vous proposons de participer à une recherche biomédicale nommée « TRIOZ ». Cette recherche a pour but de comparer l'efficacité et la tolérance de l'injection sous-conjonctivale de la triamcinolone (Kénacort Retard®) par rapport à l'injection intravitréenne de la dexaméthasone (Ozurdex®).

Le CHU de Nantes est le promoteur de cette recherche, c'est à dire qu'il en est responsable et qu'il l'organise.

COMMENT VOUS DECIDER ?

- Votre médecin-investigateur, le Dr .... vous a donné des explications. Elles sont résumées dans ce document intitulé « note d'information ». Nous vous invitons à le lire attentivement et à prendre votre temps avant de décider de votre participation à cette recherche.

- Si vous décidez de participer à cette recherche, on vous demandera de signer un formulaire de consentement. Ce formulaire sera aussi signé par votre médecin. Votre signature confirmera que vous êtes d'accord pour participer à la recherche. Votre signature est indispensable, ainsi que celle du médecin. Même après avoir signé pour donner votre accord de participation, vous garderez le droit d'interrompre à tout moment votre participation sans avoir à vous justifier.

- Si vous refusez de participer à cette étude, vous recevrez, selon l'avis de l'ophtalmologiste qui vous prend en charge, l'un ou l'autre des traitements mais les données sur l'efficacité et la tolérance de l'un ou l'autre des traitements ne seront pas colligées.

QUE SAIT-ON DEJA SUR L'ACETONIDE DE TRIAMCINOLONE (KENACORT RETARD®) PAR RAPPORT A LA DEXAMETHASONE (OZURDEX®)?

La triamcinolone (KENACORT RETARD®) est un médicament utilisé depuis de nombreuses années en ophtalmologie et en rhumatologie. Il s'agit d'un corticoïde retard, composé de cristaux qui se dissolvent très lentement. Ainsi la triamcinolone injectée dans l'œil aura un effet pendant au moins 3 mois. L'injection se fait par voie sous-conjonctivale c'est-à-dire qu'on injecte un liquide sous la conjonctive bulbaire (muqueuse qui recouvre la face postérieure des paupières et le blanc de l'œil).
L'injection se fait au moyen d'une petite aiguille très fine après anesthésie locale par un collyre anesthésique de contact. L'injection est peu douloureuse.
Bien que ce produit n'ait pas été développé pour être injecté directement dans l'œil, et qu'il n'y a pas d'Autorisation de Mise sur le Marché (AMM) pour cette injection, son usage est très répandu en ophtalmologie depuis plusieurs années pour le traitement de l'œdème maculaire.

L'OZURDEX® est un implant de très petite taille contenant un principe actif anti inflammatoire appelé la dexaméthasone, qui comme la triamcinolone est un corticoïde. La dexaméthasone est injecté directement dans l'œil, à travers la conjonctive et la sclère (blanc de l'œil) dans la cavité oculaire située en arrière du cristallin et que l'on appelle la cavité vitréenne, on parle de ce fait d'injection intravitréenne. On utilise un applicateur spécialement conçu à cet usage dont l'extrémité très fine pénètre dans l'œil anesthésié sans douleur. La piqûre se fait à un endroit où l'on peut pénétrer dans l'œil sans danger pour l'oeil. Ce médicament a l'Autorisation de Mise sur le Marché depuis 2011 pour le traitement des uvéites intermédiaires et postérieures non infectieuses compliquées d'œdèmes.

QUEL EST LE BUT DE CETTE RECHERCHE ? QUEL EST SON DEROULEMENT GENERAL ?

Les injections sous conjonctivales sont utilisées dans de nombreux services d'ophtalmologie d'hôpitaux français pour soigner l'œdème maculaire inflammatoire. Ces injections simples et peu onéreuses, faites immédiatement le jour de la consultation, semblent se montrer efficaces sur le traitement de l'œdème maculaire tant sur le plan anatomique que fonctionnel avec *a priori* peu de complications. Malheureusement il n'existe aujourd'hui aucune publication sur ces injections qui semblent pourtant simples, efficaces et bien tolérées.

De plus l'implant de dexaméthasone (OZURDEX) a obtenu l'AMM depuis 2011 pour le traitement des uvéites intermédiaires et postérieures non infectieuses compliquées d'œdèmes mais aucune étude n'a comparé l'efficacité et la tolérance de ces deux techniques dans le traitement de l'œdème maculaire inflammatoire.

L'objectif principal de cette étude est de comparer l'efficacité et la tolérance de ces 2 méthodes pour le traitement de l'œdème maculaire inflammatoire.

Quatorze centres français ont accepté de participer à cette étude : les CHU de Nantes, Lille, Lyon, Paris (La Pitié-Salpêtrière, Bicêtre, Fondation Rothschild et les Quinze-Vingts), Bordeaux, Nancy, Grenoble, Tours, Nice, Montpellier, Dijon, et 142 patients seront inclus dans cette étude, la moitié aura le traitement avec une injection sous conjonctivale de triamcinolone et l'autre moitié une injection intravitréenne de l'implant de dexaméthasone.

QU'ARRIVERA-T-IL PENDANT LA RECHERCHE ? QU'AUREZ-VOUS A FAIRE ?

Votre participation éventuelle à cette recherche durera au maximum 7 mois.

Votre accord pour participer à cette recherche demandera que vous vous engagiez pendant toute la durée de la recherche à :

- Vous rendre à toutes les visites prévues dans le protocole
  - La visite d'inclusion :

  En tout premier lieu le médecin investigateur vous expliquera oralement les objectifs, les contraintes, les bénéfices et les risques de cette étude. Si vous acceptez de participer à cette étude le médecin vous demandera vos antécédents médicaux et chirurgicaux ainsi que votre traitement actuel.

  Au cours de cette visite, voici les examens qui seront faits. Les examens numérotés de 1. à 6. sont ceux qui auraient été faits dans le cadre de la procédure standard de traitement de votre pathologie :

  1. Mesure de l'épaisseur maculaire centrale par tomographie à cohérence optique
  2. Mesure de votre acuité visuelle
  3. Mesure de la pression intraoculaire
  4. Examen du segment antérieur (+/- quantification de l'inflammation si appareil disponible)
  5. Examen du segment postérieur (fond d'œil) et photographie du fond d'œil
  6. Angiographie à la fluorescéine
  7. Mesure de la tension artérielle
  8. Questionnaire de qualité de vie
  9. Prise de sang :

- Détermination de votre hémoglobine glyquée (HbA1C) et de la glycémie à jeun pour vérifier votre taux de sucre dans le sang, les corticoïdes systémiques pouvant induire un diabète cortisoné,
- Test de grossesse pour les femmes en âge de procréer ;
- Examen et/ou analyse permettant, selon avis du médecin-investigateur, d'éliminer les pathologies qui seraient aggravées par un traitement corticoïde (si résultats non disponibles dans votre dossier).

Le médecin vous remettra en plus une carte de participation au protocole.

➢ La visite de randomisation, au cours de laquelle un tirage au sort informatisé vous placera dans l'un des deux groupes de traitement : cette visite pourra se faire en même temps que la visite d'inclusion.

Après randomisation, vous bénéficierez d'une seule injection de l'un ou l'autre des traitements c'est-à-dire soit une injection sous-conjonctivale de triamcinolone, soit une injection intra-vitréenne de dexaméthasone.

<u>Injection sous-conjonctivale de triamcinolone :</u> Vous serez installé(e) confortablement et votre œil sera insensibilisé par un collyre anesthésiant. Le médecin nettoiera votre œil avec un produit antiseptique pour diminuer le risque d'infection. L'injection ne dure que quelques minutes.

<u>Injection intravitréenne de dexaméthasone</u> : Vous revêtirez une blouse propre afin de protéger vos vêtements et l'équipe médicale vous donnera un bonnet pour vos cheveux. Vous serez ensuite allongé(e). L'œil sera insensibilisé par un collyre ou un gel anesthésiant. Le médecin nettoiera votre œil avec un produit antiseptique pour diminuer le risque d'infection. L'injection ne dure que quelques secondes et après l'injection un collyre antibiotique est de nouveau versé goutte à goutte.

Après l'injection le médecin vous demandera d'estimer la douleur de l'injection et de juger si l'injection a été tolérable, désagréable, très désagréable.

➢ Les visites de suivi seront faites mensuellement c'est-à-dire à 1 mois, 2 mois, 3 mois, 4 mois, 5 mois et 6 mois après l'injection pour contrôler l'œil.

Les examens seront :

- Mesure de l'épaisseur maculaire centrale par tomographie à cohérence optique
- Mesure de votre acuité visuelle
- Mesure de la pression intraoculaire
- Examen du segment antérieur (+/- quantification de l'inflammation si appareil disponible)
- Examen du segment postérieur (fond d'œil) et photographie du fond d'œil
- Mesure de la tension artérielle aux visites des 3 mois et des 6 mois
- Prise de sang pour la détermination de l'hémoglobine glycquée (HbA1C) et de la glycémie à jeun à la visite des 6 mois

A certaines des visites de suivi, on vous demandera aussi de remplir le questionnaire de qualité de vie. Votre traitement actuel vous sera demandé.

La visite des 6 mois sera la visite de fin d'étude.

En cas de récidive ou de persistance de votre œdème maculaire entre 3 mois et 6 mois, le médecin pourra pratiquer une injection de son choix lors de cette visite ou quelque temps après (± 30 jours). Le planning ne sera pas modifié, vous continuerez les visites restantes de l'étude.

QUELS SONT LES RISQUES ?

Le fait de participer ou non à cette recherche ne changera rien au fait que vous devrez de toute façon avoir une injection de corticoïde pour traiter votre œdème.

La prise en charge dans le cadre de cette recherche sera conforme aux recommandations dans le cadre du suivi de votre pathologie.

Evolution post-injection habituelle :

- L'oeil traité reste indolore dans la très grande majorité des cas.

- Une sensation de brûlure à la surface de l'oeil peut survenir quelques heures après l'injection. Elle est liée le plus souvent à une irritation de la cornée due au produit de désinfection et d'anesthésie. Celle-ci est sans gravité. Elle peut nécessiter l'administration d'un collyre cicatrisant.

- Il peut vous arriver de voir quelques tâches dans le champ visuel, pendant quelques heures à quelques jours, correspondant à la présence du médicament dans le vitré (le gel transparent qui remplit l'oeil derrière le cristallin). Les tâches se résorbent habituellement en quelques jours ou quelques semaines, mais peuvent persister.

Effets indésirables connus chez l'homme avec le KENACORT RETARD et l'OZURDEX :

Incidents :

L'incident le plus fréquent est sans conséquence. Il s'agit de l'hémorragie sous-conjonctivale (petite hémorragie du blanc de l'œil) qui se résorbe spontanément en quelques jours.

Complications éventuelles :

Les autres complications sont très rares et peuvent survenir malgré les précautions rigoureuses prises par le médecin. Exceptionnellement, elles peuvent prendre un caractère de gravité pouvant conduire à une perte de la vision et dans les cas les plus rares à la perte de l'œil.

- Hémorragie du vitré
- Corps flottants vitréen
- Infection

L'infection intraoculaire est un risque qui existe, comme dans n'importe quelle ponction ou acte chirurgical, malgré les précautions rigoureuses d'asepsie et la prévention par antibiotique. Le risque peut être estimé à environ 1 pour 1000 injections. L'infection intraoculaire nécessite une hospitalisation. Elle est le plus souvent traitée avec succès par des antibiotiques mais elle peut être grave pour la vision et peut, rarement, conduire à la perte de l'oeil.

L'infection se manifesterait par une baisse de la vision, des douleurs et une rougeur de l'oeil. Si vous perceviez ces symptômes dans les heures ou les jours qui suivent l'injection, contactez immédiatement votre ophtalmologiste ou le centre hospitalier le plus proche pour être pris(e) en charge sans retard.

- Hypertonie intraoculaire.

Il s'agit d'une élévation de la pression intraoculaire. Le plus souvent, il s'agit d'une hypertonie modérée, facilement contrôlée par des collyres, et qui ne sera donc aucunement fâcheuse pour la vision.

Dans de très rares cas (moins de 1 pour 1000), cette hypertonie est trop élevée malgré les collyres et une intervention chirurgicale est nécessaire pour la normaliser

- Aggravation d'une cataracte préexistante

Certains médicaments injectés, comme la cortisone, peuvent majorer l'opacification du cristallin. En cas d'injections répétées, le risque de cataracte entraînant une baisse visuelle devient important.

- Blessure accidentelle du cristallin

Elle est exceptionnelle compte tenu des précautions prises lors de l'injection.

- Décollement de la Rétine

Très rare, il se manifeste par une perte, indolore, rapidement progressive de la vision.

En cas de problème, et à tout moment dans la recherche, vous pouvez contacter votre ophtalmologiste au numéro indiqué dans le résumé (en fin de lettre).

QUELS SONT LES BENEFICES QUE VOUS POUVEZ ESPERER ?

- Bénéfices personnel thérapeutique et non thérapeutique attendu : il n'y a pas de bénéfice personnel thérapeutique et non thérapeutique attendu par rapport à la prise en charge que vous auriez eue en dehors de l'étude.

Bénéfice collectif attendu : la participation volontaire à une recherche biomédicale est utile à tous. Elle permettra de comparer les deux méthodes tant au point de vue clinique qu'au niveau du confort du patient.

QUELLES SONT LES ALTERNATIVES ?

Il n'y a pas de traitement de référence de l'œdème maculaire inflammatoire. Le traitement de référence de l'uvéite non infectieuse est les corticoïdes généraux.

QUE SE PASSERA-T-IL A LA FIN DE LA RECHERCHE, SI LA RECHERCHE S'ARRETE OU SI VOUS DECIDEZ D'INTERROMPRE VOTRE PARTICIPATION ?

La recherche peut être interrompue à tout moment :

- par les autorités de santé,
- du fait du promoteur, le CHU de Nantes : si un élément nouveau survient, le médecin-investigateur en sera informé et il vous transmettra alors les éléments susceptibles de modifier votre participation.
- du fait du médecin-investigateur, pour des raisons médicales vous concernant : il peut décider à tout moment d'arrêter l'administration du produit à l'étude (par exemple à cause d'un effet secondaire ou d'une évolution de votre état de santé) et vous en informera.
- par vous-même : si vous décidez de participer à cette recherche, il s'agira d'un acte volontaire. Vous pourrez à tout moment décider d'arrêter votre participation, sans pénalité ni préjudice. Dans ce cas, vous devez informer le médecin-investigateur de votre décision.

Quelle que soit la raison de votre interruption, le médecin-investigateur vous informera alors des mesures à suivre.

Dans tous les cas, la qualité de votre prise en charge ne sera pas diminuée.

Dans le cas où le nombre de patients prévus dans l'étude a été atteint avant que vous ne soyez inclus(e), vous pourrez finalement ne pas participer à l'étude bien qu'ayant signé un consentement. Dans ce cas précis, vous ne serez plus suivi(e) dans le cadre de l'étude et les données vous concernant seront détruites. Cela ne portera, en aucun cas, préjudice sur la qualité de votre prise en charge médicale.

AUREZ-VOUS DES FRAIS SUPPLEMENTAIRES ?

Votre participation à cette recherche n'engendrera pour vous aucun frais supplémentaire par rapport à ceux que vous auriez dans la prise en charge habituelle de cette maladie.

Si besoin, une aide à vos frais de déplacement pourra être envisagée, dans la limite d'un maximum de 100€/visite pour les visites du 1er et du 4ème mois en fonction de la distance qui sépare le lieu de visite à votre domicile et / ou la présentation d'une facture. Les autres consultations ne seront pas concernées par cette aide car elles font parties du suivi habituel.

QUELS SONT VOS DROITS PENDANT LA RECHERCHE ?

❖ SECRET PROFESSIONNEL

Le personnel impliqué dans la recherche est soumis au secret professionnel, tout comme votre médecin traitant.

❖ ACCES AUX DONNEES VOUS CONCERNANT - TRAITEMENT DES DONNEES - CONFIDENTIALITE

Dans le cadre de cette recherche, un traitement informatique de vos données personnelles va être mis en œuvre : cela permettra d'analyser les résultats de la recherche et de remplir l'objectif de la recherche.

Pour cela, les données médicales vous concernant (et les données relatives à vos habitudes de vie), seront recueillies dans un cahier électronique. Afin d'assurer leur confidentialité, ces données seront identifiées par un numéro de code et vos initiales.

L'ensemble de ces données sera transmis au Promoteur de la recherche (CHU de Nantes) et/ou aux personnes ou sociétés agissant pour son compte.

Ces données pourront également, dans des conditions assurant leur confidentialité, être transmises aux autorités sanitaires habilitées.

Elles seront susceptibles d'être exploitées dans le cadre de publications ou de communications ; dans ce cas, votre anonymat sera préservé.

Conformément aux dispositions de la loi relative à l'informatique aux fichiers et aux libertés (loi modifiée du 6 janvier 1978), de la loi n° 2018-493 du 20 juin 2018 relative à la protection des données personnelles et du Règlement (UE) 2016/679 du Parlement européen et du Conseil du 27 avril 2016 relatif à la protection des personnes physiques à l'égard du traitement des données à caractère personnel et à la libre circulation de ces données (RGPD), vous disposez d'un droit d'accès, de rectification, de portabilité et de limitation du traitement de vos données personnelles. Vous pouvez également introduire une réclamation auprès d'une autorité de contrôle (CNIL pour la France).

Vous pouvez également accéder directement ou par l'intermédiaire d'un médecin de votre choix à l'ensemble de vos données médicales en application des dispositions de l'article L 1111-7 du Code de la Santé Publique.

Vos données seront conservées tout au long de la recherche. Après la fin de la recherche, les données seront archivées pour une durée conforme aux dispositions réglementaires, puis détruites.

Pour en savoir plus ou exercer vos droits concernant vos données, voir vos contacts en annexe 1.

❖ ACCES AUX RESULTATS GLOBAUX DE LA RECHERCHE

A la fin de la recherche biomédicale, et à votre demande, vous pourrez être informé(e) par le médecin-investigateur des résultats globaux de cette recherche (dès qu'ils seront disponibles).

QUELLES SONT VOS OBLIGATIONS PENDANT LA RECHERCHE ?

❖ VOS OBLIGATIONS

Vous devez informer votre médecin investigateur de tous les médicaments que vous prenez.

Vous devez aussi l'informer immédiatement de tout effet indésirable éventuellement rencontré au cours de votre participation à la recherche.

Vous devez vous rendre aux visites prévues.

❖ PROTECTION SOCIALE

Pour pouvoir participer à cette recherche vous devez être affilié(e) ou bénéficier d'un régime de sécurité sociale *(CMU acceptée)*.

❖ MODALITES DE PARTICIPATION A UNE AUTRE RECHERCHE

Pendant votre participation à la recherche vous ne pourrez pas participer à une autre recherche clinique.

LE CADRE REGLEMENTAIRE

Cette recherche est conforme :

- Aux articles L. 1121-1 à L. 1126-7 du code de la santé publique relatifs aux recherches biomédicales,

- A la loi « Informatique et Libertés » du 6 janvier 1978 modifiée et la loi n° 2018-493 du 20 juin 2018 relative à la protection des données personnelles

- au Règlement (UE) 2016/679 du Parlement européen et du Conseil du 27 avril 2016 relatif à la protection des personnes physiques à l'égard du traitement des données à caractère personnel et à la libre circulation de ces données (RGPD).
(Vous pouvez retrouver tous ces textes sur le site http://www.legifrance.gouv.fr)

Conformément aux dispositions réglementaires :

➢ Le CHU de Nantes organise cette recherche en tant que « promoteur ». Il a souscrit un contrat d'assurance garantissant sa responsabilité civile et celle de tout intervenant auprès de la compagnie HDI-GERLING (contrat n°0100755914039-150010-10998).

Cette recherche a reçu l'avis favorable du Comité de Protection Ouest II le 01/09/2015. La recherche a aussi reçu l'autorisation de l'ANSM (Agence Nationale de Sécurité du Médicament), le 25/08/2015.

<u>EN RESUME…</u>

Votre participation à cette recherche est libre. Vous pouvez refuser de participer à cette recherche.

De plus, vous pouvez à tout moment vous retirer de cette recherche, sans préjudice.

Si vous décidez de refuser de participer à la recherche ou si vous décidez d'arrêter votre participation pendant la recherche :

- **cela n'aura aucune conséquence sur la qualité des soins qui vous seront donnés**
- **vous devez simplement en informer votre médecin-investigateur.**

Lorsque vous aurez lu cette note d'information et obtenu les réponses aux questions que vous vous posez en interrogeant le médecin-investigateur, il vous sera proposé, si vous en êtes d'accord, de donner votre consentement écrit en signant le formulaire préparé à cet effet.

Votre participation à la recherche, au cas où vous donneriez votre accord, ne pourra vous être confirmée qu'à la condition que vous remplissiez tous les critères d'inclusion pour participer à cette recherche.

Vous pouvez prendre votre temps avant de nous donner votre réponse.

Au cours de ce délai de réflexion, vous pouvez bien entendu continuer par téléphone à poser toutes les questions que vous souhaitez à votre médecin (Dr/Pr…………………….. tél………………………..).

Nous vous prions d'agréer, Madame, Mademoiselle, Monsieur, l'expression de nos sentiments les plus respectueux.

Pr Michel Weber, médecin coordonnateur,

et toute l'équipe médicale en charge de cette recherche

Vous devez conserver un exemplaire de ce document.

ANNEXE 1 :
LES CONTACTS IMPORTANTS

Pour toute question concernant l'étude, retrait de consentement, ou pour exercer vos droits concernant vos données (accès rectification, etc…) :

Le médecin-investigateur coordonnateur de la recherche :

Professeur Michel Weber

Service d'Ophtalmologie

✉ 1 place Alexis Ricordeau, 44093 Nantes cedex

☎+ 33 (0) 2 40 08 34 06

Pour toute question générale sur la protection de vos données :

Le promoteur de la recherche, responsable du traitement :

CHU de Nantes, direction de la recherche

5 allée de l'Ile Gloriette, 44093 NANTES Cedex 1

Le Délégué à la protection des Données (DPO) :

vosdonneespersonnelles@chu-nantes.fr

<table>
<tr><td rowspan="2">CENTRE HOSPITALIER UNIVERSITAIRE DE NANTES</td><td rowspan="3">Formulaire de consentement à une recherche
« Comparaison de l'efficacité et de la tolérance d'une injection sous-conjonctivale de triamcinolone et d'un implant intravitréen de dexaméthasone sur l'œdème maculaire inflammatoire.»
Promoteur : CHU de Nantes
Réf : RC15_0056 N° EudracT / n° d'enregistrement : 2015-000468-33</td></tr>
<tr></tr>
<tr><td>Version n°4
Date : 16/07/2018</td></tr>
</table>

Je soussigné(e)

M^e^, Mlle, M. *(rayer les mentions inutiles)* (*prénom*, NOM) ……………………………………………………………………………………………………,

Date de naissance : …../……/……..
accepte librement et volontairement de participer à la recherche référencée ci-dessus, coordonnée par le Professeur Weber et organisée par le CHU de Nantes, promoteur de la recherche.

Etant entendu que :

- Le médecin qui m'a informé(e) et a répondu clairement à toutes mes questions, m'a précisé que ma participation est libre et que je peux me retirer de la recherche à tout moment.
- J'atteste ne pas faire l'objet de mesure de protection (tutelle, curatelle, sauvegarde de justice), en outre je confirme être affilié(e) ou bénéficier d'un régime de sécurité sociale.
- Il m'a été préalablement remis une note d'information sur cette recherche précisant son but, sa méthodologie, ses bénéfices attendus et ses risques prévisibles.
- Avant de participer à cette recherche, j'ai bénéficié d'un examen médical dont les résultats m'ont été communiqués.
- Je pourrai avoir communication par le médecin, au cours ou à l'issue de la recherche, des informations qu'il détient concernant ma santé.
- Si le nombre de patients prévus dans l'étude a été atteint, je pourrais finalement ne pas être inclus(e) dans l'étude bien qu'ayant signé un consentement. Dans ce cas précis, je ne serai plus suivi(e) dans le cadre de l'étude et les données me concernant ainsi que les échantillons sanguins le cas échéant seront détruits. Cela ne portera, en aucun cas, préjudice sur la qualité de ma prise en charge médicale.
- Je suis parfaitement conscient(e) que je peux retirer à tout moment mon consentement à ma participation à cette recherche et cela quelles que soient mes raisons et sans supporter aucune responsabilité, mais je m'engage dans ce cas à en informer le médecin. Le fait de ne plus participer à cette recherche ne portera pas atteinte à mes relations avec ce médecin, ni à la qualité des soins qui me seront donnés.
- J'accepte que mon médecin traitant soit informé de ma participation à la recherche :
  ☐ Oui, j'accepte ☐ Non, je refuse
- Je pourrai à tout moment demander des informations complémentaires au médecin.
- Si je le souhaite, à son terme, je serai informé(e) par le médecin des résultats globaux de cette recherche.
- Mon consentement ne décharge en rien le médecin et le promoteur de l'ensemble de leurs responsabilités et je conserve tous mes droits garantis par la loi.
- Je ne pourrai pas participer à une autre recherche biomédicale pendant toute ma participation à cette recherche.
- J'accepte que les données enregistrées à l'occasion de cette recherche puissent faire l'objet d'un traitement informatisé par le promoteur ou pour son compte, et j'atteste avoir été informé(e) de tous mes droits concernant mes données personnelles selon les modalités décrites dans la note d'information en vigueur, qui m'a été transmise pour ce protocole.

<table>
<tr><td>Date :</td><td colspan="2">Le cas échéant : Attestation du consentement en cas d'impossibilité d'expression écrite de la personne qui se prête à la recherche biomédicale (précision du titre de la personne)</td></tr>
<tr><td><u>Signature du patient :</u></td><td>Date :
Prénom NOM :</td><td><u>Signature :</u></td></tr>
</table>

<table>
<tr><td colspan="2">Signature du médecin qui atteste avoir pleinement expliqué à la personne signataire le but, les modalités ainsi que les risques potentiels de la recherche.</td></tr>
<tr><td>Date :</td><td><u>NOM et Signature :</u></td></tr>
</table>

<table>
<tr><td colspan="2">En cas de retrait de consentement (cocher la case correspondante)</td></tr>
<tr><td>☐ J'accepte que mes données soient analysées</td><td>☐ Je ne veux pas que mes données soient analysées</td></tr>
</table>

Ce document est à réaliser en 2 exemplaires originaux : le premier doit être conservé par l'investigateur et le deuxième est remis à la personne donnant son consentement. En cas de duplicata, l'original est conservé par l'investigateur et une copie est remise à la personne ayant donné son consentement.
