# Supplementary material for: Comparison of two techniques used in routine care for the treatment of inflammatory macular oedema, subconjunctival triamcinolone injection and intravitreal dexamethasone implant: medical and economic importance of this randomized controlled trial
Source: Trials. 2020 Feb 10;21:159. doi: 10.1186/s13063-020-4066-0 (PMC7011383; doi:10.1186/s13063-020-4066-0)
Supplement: Supplementary file 5 — Additional file 5. SPIRIT 2013 checklist: recommended items to address in a clinical trial protocol and related documents. [file 13063_2020_4066_MOESM5_ESM.docx]

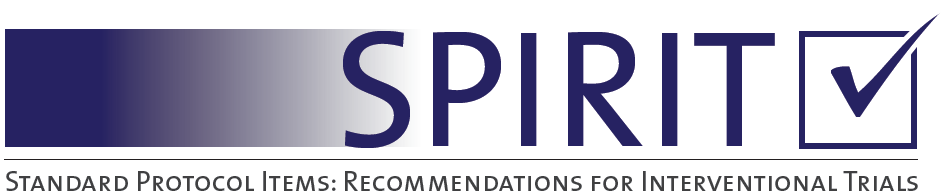


SPIRIT 2013 Checklist: Recommended items to address in a clinical trial protocol and related documents*

| Section/item | ItemNo | Description |
| --- | --- | --- |
| **Administrative information** | | |
| Title | 1 | Descriptive title identifying the study design, population, interventions, and, if applicable, trial acronym🡺see pg1 line 1 in Title |
| Trial registration | 2a | Trial identifier and registry name. If not yet registered, name of intended registry 🡺see pg3 line 68 in Abstract – Trial Registration |
|  | 2b | All items from the World Health Organization Trial Registration Data Set  **Item 1** 🡺 see pg3 line 68 Abstract – Trial Registration  **Item 2** 🡺 Ditto item 1  **Item 3** 🡺 *Not Applicable*  **Item 4** 🡺 Pg 24 line 624 in Ethical, regulatory and dissemination aspects and pg28 line 713 in Funding  **Item 5** 🡺 Pg 23 line 596 in Ethical, regulatory and dissemination aspects  **Item 6** 🡺*Not Applicable*  **Item 7** 🡺 Pg 1 Authors from Sponsor Department of CHU Nantes  **Item 8** 🡺 Pg 1 corresponding Author  **Item 9** 🡺 Pg 28 line 715 in Tittle of the trial in Funding  **Item 10** 🡺 Ditto item 9  **Item 11** 🡺 Pg 9 line 202 in Study Population - Description of the population and Pg 9 line 209 in Study Design - Recruitment for the trial  **Item 12** 🡺 Pg 5- 8 Background and Pg 10 line 209 in Study Design - Recruitment for the trial  **Item 13** 🡺 Pg 9 line 187 in Study design  **Item 14** 🡺see Additional File 2 and Pg 9 line 209 in Recruitment for the trial  **Item 15** 🡺 Pg 9 line 187 in Study design  **Item 16** 🡺 Pg 26 in Trial Status  **Item 17** 🡺 Pg 20 in Sample size  **Item 18** 🡺 Ditto item 16  **Item 19** 🡺 Pg 16 line 378 in Outcome  **Item 20** 🡺 Pg 16 line 395 in Outcome  **Item 21** 🡺 Pg 23 line 581 in Ethical, regulatory and dissemination aspects  **Item 22** 🡺 Pg 26 line 662 in Trial Status  **Item 23** 🡺 Not Applicable  **Item 24** 🡺 Pg 23 line 581 in Ethical, regulatory and dissemination aspects |
| Protocol version | 3 | Date and version identifier 🡺see pg 23 line 595 in Ethical, regulatory and dissemination aspects |
| Funding | 4 | Sources and types of financial, material, and other support 🡺see pg28 line 714 Funding |
| Roles and responsibilities | 5a | Names, affiliations, and roles of protocol contributors 🡺see pg1 line 7 Authors and pg 29 line 727 in Authors’ contribution |
|  | 5b | Name and contact information for the trial sponsor 🡺see Pg 1 Authors from Sponsor Department of CHU Nantes |
|  | 5c | Role of study sponsor and funders, if any, in study design; collection, management, analysis, and interpretation of data; writing of the report; and the decision to submit the report for publication, including whether they will have ultimate authority over any of these activities 🡺see pg 23 line 596 in Ethical, regulatory and dissemination aspects |
|  | 5d | Composition, roles, and responsibilities of the coordinating centre, steering committee, endpoint adjudication committee, data management team, and other individuals or groups overseeing the trial, if applicable (see Item 21a for data monitoring committee) 🡺see pg 22 line 565 in SAE Reporting and pg 24 line 603 in Ethical, regulatory and dissemination aspects |
| Introduction |  |  |
| Background and rationale | 6a | Description of research question and justification for undertaking the trial, including summary of relevant studies (published and unpublished) examining benefits and harms for each intervention 🡺see pg 5 – 8 Background |
|  | 6b | Explanation for choice of comparators 🡺 ditto item 6a |
| Objectives | 7 | Specific objectives or hypotheses 🡺see pg 8 line 179 in Background and pg 15 line 351 in Objectives |
| Trial design | 8 | Description of trial design including type of trial (eg, parallel group, crossover, factorial, single group), allocation ratio, and framework (eg, superiority, equivalence, noninferiority, exploratory) 🡺see pg9 line 187 in Study design |
| Methods: Participants, interventions, and outcomes | | |
| Study setting | 9 | Description of study settings (eg, community clinic, academic hospital) and list of countries where data will be collected. Reference to where list of study sites can be obtained 🡺see pg 9 line 201 in Description of the population |
| Eligibility criteria | 10 | Inclusion and exclusion criteria for participants. If applicable, eligibility criteria for study centres and individuals who will perform the interventions (eg, surgeons, psychotherapists) 🡺see Additional File 2 and pg 10 line 208 in Recruitment for the trial |
| Interventions | 11a | Interventions for each group with sufficient detail to allow replication, including how and when they will be administered 🡺see figure 3 and pg 20 line 516 in Randomization |
|  | 11b | Criteria for discontinuing or modifying allocated interventions for a given trial participant (eg, drug dose change in response to harms, participant request, or improving/worsening disease) 🡺see pg 14 line 340 in Study Schedule |
|  | 11c | Strategies to improve adherence to intervention protocols, and any procedures for monitoring adherence (eg, drug tablet return, laboratory tests) 🡺see pg 13 line 318 in Study Schedule |
|  | 11d | Relevant concomitant care and interventions that are permitted or prohibited during the trial 🡺see pg 12 line 287 in Treatment visit |
| Outcomes | 12 | Primary, secondary, and other outcomes, including the specific measurement variable (eg, systolic blood pressure), analysis metric (eg, change from baseline, final value, time to event), method of aggregation (eg, median, proportion), and time point for each outcome. Explanation of the clinical relevance of chosen efficacy and harm outcomes is strongly recommended 🡺see pg 16 to 18 in Outcomes |
| Participant timeline | 13 | Time schedule of enrolment, interventions (including any run-ins and washouts), assessments, and visits for participants. A schematic diagram is highly recommended (see Figure) 🡺see Figure 3 |
| Sample size | 14 | Estimated number of participants needed to achieve study objectives and how it was determined, including clinical and statistical assumptions supporting any sample size calculations 🡺see pg 20 in Sample size |
| Recruitment | 15 | Strategies for achieving adequate participant enrolment to reach target sample size 🡺see pg 9 line 206 in Study Population |
| **Methods: Assignment of interventions (for controlled trials)** | | |
| Allocation: |  |  |
| Sequence generation | 16a | Method of generating the allocation sequence (eg, computer-generated random numbers), and list of any factors for stratification. To reduce predictability of a random sequence, details of any planned restriction (eg, blocking) should be provided in a separate document that is unavailable to those who enrol participants or assign interventions 🡺see pg 20,21 in Randomization |
| Allocation concealment mechanism | 16b | Mechanism of implementing the allocation sequence (eg, central telephone; sequentially numbered, opaque, sealed envelopes), describing any steps to conceal the sequence until interventions are assigned 🡺see pg 21 line 518 in Randomization |
| Implementation | 16c | Who will generate the allocation sequence, who will enrol participants, and who will assign participants to interventions 🡺see pg 21 line 528 in Randomization |
| Blinding (masking) | 17a | Who will be blinded after assignment to interventions (eg, trial participants, care providers, outcome assessors, data analysts), and how 🡺see pg 9 line 191 in Study Design and pg 21 line 524 in Randomization |
|  | 17b | If blinded, circumstances under which unblinding is permissible, and procedure for revealing a participant’s allocated intervention during the trial 🡺see pg 9 line 191 in Study Design |
| **Methods: Data collection, management, and analysis** | | |
| Data collection methods | 18a | Plans for assessment and collection of outcome, baseline, and other trial data, including any related processes to promote data quality (eg, duplicate measurements, training of assessors) and a description of study instruments (eg, questionnaires, laboratory tests) along with their reliability and validity, if known. Reference to where data collection forms can be found, if not in the protocol 🡺see pg 17-18 in Measures used to determine the outcomes |
|  | 18b | Plans to promote participant retention and complete follow-up, including list of any outcome data to be collected for participants who discontinue or deviate from intervention protocols 🡺see pg13 line 313 |
| Data management | 19 | Plans for data entry, coding, security, and storage, including any related processes to promote data quality (eg, double data entry; range checks for data values). Reference to where details of data management procedures can be found, if not in the protocol 🡺see pg 23 line 596 in Ethical, regulatory and dissemination aspects |
| Statistical methods | 20a | Statistical methods for analysing primary and secondary outcomes. Reference to where other details of the statistical analysis plan can be found, if not in the protocol 🡺see pg 18-20 in Statistical methods |
|  | 20b | Methods for any additional analyses (eg, subgroup and adjusted analyses) 🡺see pg 19 line 463 in Statistical methods |
|  | 20c | Definition of analysis population relating to protocol non-adherence (eg, as randomised analysis), and any statistical methods to handle missing data (eg, multiple imputation) 🡺see pg 20 line 494 in Statistical methods |
| **Methods: Monitoring** | | |
| Data monitoring | 21a | Composition of data monitoring committee (DMC); summary of its role and reporting structure; statement of whether it is independent from the sponsor and competing interests; and reference to where further details about its charter can be found, if not in the protocol. Alternatively, an explanation of why a DMC is not needed 🡺see pg 22 line 565 in SAE Reporting |
|  | 21b | Description of any interim analyses and stopping guidelines, including who will have access to these interim results and make the final decision to terminate the trial 🡺see pg 23 line 573 in SAE Reporting |
| Harms | 22 | Plans for collecting, assessing, reporting, and managing solicited and spontaneously reported adverse events and other unintended effects of trial interventions or trial conduct 🡺see pg 22 line 560 in SAE Reporting |
| Auditing | 23 | Frequency and procedures for auditing trial conduct, if any, and whether the process will be independent from investigators and the sponsor 🡺see pg 24 line 615 |
| Ethics and dissemination | | |
| Research ethics approval | 24 | Plans for seeking research ethics committee/institutional review board (REC/IRB) approval 🡺see pg 23 line 587 in Ethical, regulatory and dissemination aspects |
| Protocol amendments | 25 | Plans for communicating important protocol modifications (eg, changes to eligibility criteria, outcomes, analyses) to relevant parties (eg, investigators, REC/IRBs, trial participants, trial registries, journals, regulators) 🡺see pg 23 in Ethical, regulatory and dissemination aspects |
| Consent or assent | 26a | Who will obtain informed consent or assent from potential trial participants or authorised surrogates, and how (see Item 32) 🡺see pg 10 line 223 in Trial recruitment |
|  | 26b | Additional consent provisions for collection and use of participant data and biological specimens in ancillary studies, if applicable The participants data won’t be used for other studies as written in information file (Additional file 4) |
| Confidentiality | 27 | How personal information about potential and enrolled participants will be collected, shared, and maintained in order to protect confidentiality before, during, and after the trial 🡺see pg 23 in Ethical, regulatory and dissemination aspects |
| Declaration of interests | 28 | Financial and other competing interests for principal investigators for the overall trial and each study site 🡺see pg 28 line 709 in Declaration of Competing Interest |
| Access to data | 29 | Statement of who will have access to the final trial dataset, and disclosure of contractual agreements that limit such access for investigators🡺see pg 23 in Ethical, regulatory and dissemination aspects |
| Ancillary and post-trial care | 30 | Provisions, if any, for ancillary and post-trial care, and for compensation to those who suffer harm from trial participation 🡺see pg 23 line 601 in Ethical, regulatory and dissemination aspects |
| Dissemination policy | 31a | Plans for investigators and sponsor to communicate trial results to participants, healthcare professionals, the public, and other relevant groups (eg, via publication, reporting in results databases, or other data sharing arrangements), including any publication restrictions 🡺see pg 24 line 619 in Ethical, regulatory and dissemination aspects |
|  | 31b | Authorship eligibility guidelines and any intended use of professional writers ditto item 31a |
|  | 31c | Plans, if any, for granting public access to the full protocol, participant-level dataset, and statistical code 🡺see pg 24 line 598 in Ethical, regulatory and dissemination aspects |
| Appendices |  |  |
| Informed consent materials | 32 | Model consent form and other related documentation given to participants and authorised surrogates 🡺 see Additional file 4 |
| Biological specimens | 33 | Plans for collection, laboratory evaluation, and storage of biological specimens for genetic or molecular analysis in the current trial and for future use in ancillary studies, if applicable Blood tests validate inclusion criteria and, as in common practice, control the onset of treatment-related diabetes. They're not going to be stored as a biocollection. |

*It is strongly recommended that this checklist be read in conjunction with the SPIRIT 2013 Explanation & Elaboration for important clarification on the items. Amendments to the protocol should be tracked and dated. The SPIRIT checklist is copyrighted by the SPIRIT Group under the Creative Commons “[Attribution-NonCommercial-NoDerivs 3.0 Unported](http://www.creativecommons.org/licenses/by-nc-nd/3.0/)” license.
